# Austin District Medical Society: Amputation of Cervix Uteri, Discussion On; New Operation for Hernia; Empyema, Discussion On; Typhlitis, Discussion on, Etc.

**Published:** 1891-07

**Authors:** T. J. Bennett, J. W. McLaughlin

**Affiliations:** Secretary; President


### Society Notes.

Reported for the Official Organ by Seth M. Morris. M. D.

### Austin District Medical Society.

This body held its regular quarterly meeting in Medical Hall, Austin, June 25, with a full attendance from surrounding counties. Dr. J. W. McLaughlin, President, in the chair at morning session; Vice-President Ellison presided in the afternoon. Seven new members were admitted, to-wit: Drs. W. P. Means, Cotton Gin; Mat. M. Smith, Seth M. Morris, W. J. Matthews and R. E. Shelton, Austin, and J. H. Burleson, Lampasas. It was one of the most interesting and practically valuable meetings yet held. The Society is literally on a boom, and now numbers nearly one hundred working members.

Dr. J. Cummings opened the proceedings by a paper on "Amputation of the Cervix," and gave a number of cases in which he claimed the operation--performed mainly for elongation, with dysmenorrhœa and sterility—had been attended with the best results; also gave cases of operation for other causes, as ulcers, fissure, etc.

DISCUSSION.

Dr. G. W. Christian said the operation as performed by Sims has its legitimate sphere, but is not justified in laceration or ulceration; while in cancer there could be no doubt of its indication. In deep laceration, or in extensive cicatrices, the operation of Emmet is preferable. He had found it successful in relieving all trouble, local and nervous. In conical os, dilation should be tried before amputation is resorted to.

Dr. Hadra said, when the cervix is diseased the indication is clear for its removal, if it can in no other way be relieved. The conditions which may render an amputation necessary, are too numerous to be enumerated, and the surgeon must be the judge. But here he would enquire, what constitutes an amputation of the cervix? Much or little tissue might be removed, and in either case it would be called an amputation. For instance, for an ulcer, it might do to slice off a thin piece, and that would be classed as an amputation; if the entire cervix be removed, it is no more than an amputation. This is a most dangerous operation. In his judgment, more so than the removal of the entire uterus, because in the latter you have access to, and control of bleeding vessels, while in the former you cannot always stop hemorrhage. Amputation of the cervix alone should not be done for cancer, the entire womb should be removed, before you can be sure you have extirpated the disease. Amputation for any cause is a grave operation, and should not be lightly done. If the cause is sufficient, of course you have to operate. Emmet's operation is a species of amputation. The trouble is afterwards, as occlusion is apt to result. It is recommended to "cover the stump with mucous membrane." That is easier said than done. It looks well when well done, and just after operation, but in three or four days we find the stitches tear out. That is the experience of most surgeons, and occlusion will result and give trouble for years. Those who have not had such experience are to be congratulated. I do not operate now, as formerly, for almost everything; only when I think it is absolutely necessary; for instance, in malignant diseases, total extirpation is the only hope of cure. The New York surgeons are unsuccessful in extirpation of the womb and appendages, but in malignant disease the general statistics make a favorable showing for the operation. It is, if properly done, safer and less dangerous than amputation of the cervix. There are few cases—leaving out malignant disease—which cannot be relieved by other means, and the field for this operation (amputation cervix) is narrowed down to a few cases. We older men have learned that it is not necessary to operate on the cervix as often as before.

Dr. Denton records his emphatic opposition to the operation as described by Dr. Cummings. The causes asigned by Dr. C. (elongated os, conical os, ulceration, laceration, anteflexion, retroflexion, stenosis, etc.) were, twenty-five years ago, thought sufficient to warrant the amputation of more or less of the os, but in the conditions related by Dr. Cummings the operation is not justified. If you have a ragged cervix and cannot heal it, will it be any better after an amputation? You undertake to cover the stump with mucous membrane, as in circumcision, and, as Dr. Hadra says, you will find retraction, and the stitches will pull out. Emmet says removal of cervix is not called for except for malignant disease. Few will be bold enough to say laceration justifies it. In my judgment, amputation as described by Dr. C. in the cases cited, is not justified. In later works you will find authors say removal of entire organ (for malignant disease) is, as Dr. H. points out, to be preferred. The reason is very clear: you cannot know, in amputation, whether or not you have removed the disease. Dr. Cummings quotes Sims as condemning the operation in his after years. I don' believe the operation is ever called for.

Dr. Wooten said that his views had been so fully expressed by Drs. Hadra and Denton, that he had little to add. He is conservative,—and in a long life of active practice he had never found it necessary to excise the os uteri for any cause,—never saw a case where he thought it was justified, much less advisable. Especially in cancer is it to be condemned, for the reasons given by Drs. H. and D.—the remaining parts will re-develop the disease. If you are sure the cancer is confined to the uterus, and does not extend to adjacent parts, extirpate the entire organ, of course. As to an ulcer "which may become cancerous," as I heard just now, that is sheer nonsense. An ulcer cannot "run into cancer." To amputate for misplacement, is a great mistake; equally so sometimes for elongation, for elongation is often the result of misplacement, and not the cause. We see no more reports now of amputation for such; and with the general profession it is a rare condition which is thought to call for amputation of cervix. Saw a case once operated on for elongated cervix (thought to prevent conception) when the operation was new, and the woman aborted next day. This shows the necessity of great caution.

Dr. J. A. Davis was surprised to hear Dr. Hadra recommend total excision of womb for cancer of the neck. Cannot see why the whole womb should be removed. Would remove the smallest possible amount of tissue compatible with extent of disease.

Dr. Morris was not posted as to statistics of removal of womb for cancer, but in his own experience, in true cancer it is only safe to extirpate the womb. We know in cancer of the breast the disease will return; so in the uterus, and in the latter you have a better chance to get rid of the disease by removal of organ than in cancer of breast. Concurs with Drs. Wooten, Hadra and Denton in their views. It is a question each must decide for himself: Can you relieve such and such a condition of os or uterus or bladder by ordinary means, before resorting to amputation? What means are best? There are some cases of granulation where excision might do good, as cauterization with nitric acid sometimes leaves an ulcer. What are we to gain by amputation? what are the statistics? what are the chances for the patient? are all to be gravely considered. Has never made an amputation of cervix for malignant disease, and does not know of a cure by these piece-meal operations. Would remove the womb for cancer of the cervix.

Dr. Davis thought Dr. Cumming's paper a good one, but that the author goes too far, especially when he speaks of "ulcers running into cancers." The Doctor clearly did not mean it.

Dr. Cummings remarked that cancer often begins as an ulcer, and where a diathesis exists no one could tell, even with microscope, if an ulcer would become a cancer.

Dr. Hadra reminded Dr. Davis, replying to his first remarks, that in cancer of breast it is necessary to remove adjacent glands (axilla) to ensure safety; so in womb. If one could be sure cancer is confined to the neck, then amputation would be sufficient; but who can tell?

Dr. M. A. Taylor thought the discussion had gone beyond its proper bounds; the question was on the justifiability of amputating the cervix for conditions enumerated by the paper. He operated many times, with good results. Sims' operation was devised for sterility, and his thoughts turned to elongated cervix as a cause of sterility, and began amputating elongated cervix. The profession is abandoning the operation, as they find simpler means will relieve many cases where operation would formerly have been resorted to. Would, though, if he had an ulcer he could not heal, excise it, just as he would do in a suppurating bubo which he could not heal. Would surely excise a syphilitic lucer, if sure there was no cancer.

Dr. Wooten replied to Dr. Taylor as to ulcer of the cervix. In thirty years experience with them, he had never seen one he could not heal; never thought of amputating the cervix for one. Has used caustics, and always with success. Of course, the syphilitic condition should be destroyed, and inordinate granulations removed; it will then heal. Even this (syphilitic) form of ulcer does not require amputation.

Dr. Taylor: "You curette a syphilitic ulcer; which is easier, curetting or amputation? They amount to same thing, if a sharp currette be skillfully used; and if the curette be dull you had better amputate and be done with it." [Why not get a sharp curette?—Ed.]

Dr. Christian: "Dr. Hadra holds that total extirpation of uterus is less dangerous than amputation of os; if so in cancer of the womb—and he deferred to Dr. Hadra's experience, he would extirpate; but in his experience removal of womb for cancer was an exceedingly grave operation; and surely, no surgeon would resort to it except as a *dernier ressort*." The statistics he has seen show a heavy mortality, whereas, for amputation of cervix the death-rate is very small. [How about the cures?] If I had any doubt of getting all the disease by amputating I would resort to total extirpation. But we cannot always have our way. We have sometimes to be governed by the will of the patient or family. A case presents; we advise entire removal of uterus (cancer of neck); family object, and insist on a partial operation. Who will say that removal of cervix for cancer has not prolonged life? Who would send this patient away without operating? without removing as much as you can, because she will not consent to the major operation? I had such experience, and gave my patients several months of relief. They stand the amputation of cervix well. My patients have done well, gained strength and appetite, and no doubt life has been prolonged. As to amputating cervix for other causes, I believe the operation has a legitimate field, but that it is abused there is no doubt.

What will you do with a long cervix, protruding from the body?

Drs. Denton and Davis never saw such cases.

Dr. Christian: "But they do exist."

Dr. Poyner: "Never resorted to cutting the os in his life; never saw a case he could not relieve without." [Referring to minor troubles—not cancer.—Ed.]

Dr. Morris [the Nestor of Austin profession.—Ed.]: "I would advise all to consult statistics; when a paper is to be read and is announced—read up on the subject and be able to state facts. Do not rely opon your experience solely; be able to give reliable data, so that the younger men may not be misled. Our experiences are so varied that that of no one man may safely be taken as an unerring guide."

Dr. Cummings, in closing, said: "Statistics in amputation of cervix for cancer give five per cent. of deaths. Operators had tried in vain to bring total extirpation up to this work, and in New York the profession is *blue* over the rapid return of the disease after the total removal of the uterus. Cancer is of local origin and I differ with Dr. Wooten. Ulcers may become cancerous. I knew what Dr. Denton's criticism would be. He would remind him of the enmity that existed between Sims and Emmet, which would account for their difference. The gentlemen have generally misconstrued my remarks. In speaking of amputating the cervix for laceration, I meant deep laceration, where cicatix existed and which could not be relieved by other means. Also in ulceration, I referred to the obstinate and unyielding only. But I observe no speaker has referred to the relief to the bladder unquestionably afforded by amputation in certain cases."

Dr. Denton: "In what class of lacerations would you operate?"

Dr. Cummings: "I have just stated, in deep or cicatrized, which will not yield to other means."

Dr. Denton, "Did you ever see one?"

Dr. Commings: "No; but others have."*

*[Dr. Wooten was to have had a paper on Pessaries, but owing to press of business, had not been able to prepare it, but he read a brief outline of the subject, giving the conditions in which pessaries are to be used, supplementing it with a full and learned discussion of the subject. This opened the discussion, which was very full, and on motion Dr. Wooten was requested to reduce his remarks to writing for publication. As we hope to have this valuable paper for September, we omit here the discussion, and will publish it with the paper in September.—Ed.]

Dr. Leake read a paper on Hysteria, which will be printed in the next issue of the JOURNAL. Discussion omitted till then.

NEW OPERATION FOR HERNIA.

Dr. Hadra next read a paper in which he advocated a new operation, designed by himself, for the radical cure of inguinal hernia. The points aimed at especially in his operation are to entirely obliterate that depression in the abdominal wall near the internal abdominal ring, which serves as a kind of guide to that opening for the intestine; and secondly, to secure greater surface for adhesion, so that the resistance offered to the hernia by the abdominal wall, would be greater than is the case after other operations.

The paper was highly complimented and several expressed their faith in the operation as the surgical procedure of the future.

Dr. Samuel Cunningham then read a paper on Empyema, in which he reported an interesting case that occurred in his practice.

Empyema or Suppurative Pleuritis, were the terms, he said, invariably employed to denote an accumulation of purulent fluid in the plural cavity. The etiolgy was given as traumatic, or idiopathic, but most frequently it was secondary to pleurisy with effusion, or to pneumonia. The disease is most generally unilateral. Among the notable symptoms were mentioned dyspnœa, threatened syncope at times, especially when the patient turns on the affected side, increase in the frequency of the heart, fever of a low grade with occasional exacerbations, and a troublesome, usually dry, and at times very fatiguing cough. Diminution of the respiratory movement on the affected side, and an œdema of the tissues, which pit on pressure if the effusion is purulent. The patient lies on the affected side. The physical signs are given as dullness on percussion, an indistinct respiratory murmur, sometimes bronchophony or œgophony, and if the effusion is large, a displaced heart. The exploring needle reveals the true condition of affairs. Thoracentesis, he considered the only rational treatment. The prognosis was grave and rendered worse by delay. Aspiration of a considerable part of the fluid before the operation of thoracentesis was done, was advised in order to obviate the possible dangers arising from a too sudden emptying of the cavity. The injection of antiseptics into the cavity was recommended. The patient, white aged 26, was suddenly seized with a violent chill on February 6, 1891. He was first seen on February 8, and the diagnosis of pluro-pneumonia of right side was made. Improvement took place under treatment and in ten days his condition promised rapid recovery. At the end of three weeks he relapsed with the symptoms of a typical pneumonia. Treatment was symptomatic. Fever of a typhoid character continued for thirty days, but during that time careful and repeated examinations failed to detect fluid. A few days afterwards the symptoms of empyema appeared. After consultation aspiration was decided upon. Seven pints of thin sero-fibrinous fluid were drawn off through the sixth interspace latterly. The aspiration was repeated twice, at intervals of five or six days, removing a quart and a half gallon respectively. After each aspiration the cavity was irrigated with 109% hydrogen peroxid, and at the last operation a tube was inserted and the cavity washed out, every forty-eight hours, for several days. The tube accidentally came out and was not replaced. Aspiration again removed a quantity of pus, and the cavity was again irrigated. Eight days later a trocar was introduced through the eighth inter-costal space posteriously, and by means of the surgical pump a gallon of pus was removed. The cavity was then thoroughly washed out with carbolic acid (%?), and the irrigations repeated, forty-eight to seventy-two hours apart, for three weeks. The tube is still kept in place, but the opening has become fistulous and the patient removes the tube at will. There is now a slight discharge only, and the patient is improving in general health. Lately he has been on potassium iodid., mercuric chlorid (as an alterative), and iron and quinine as tonics.

DISCUSSION.

Dr. Wooten endorsed the treatment of aspiration, drainage, and then irrigation of the cavity. He was of the opinion that the danger of admitting air were overestimated, but still thought it was best to not leave the opening entirely exposed.

Dr. Mathews said, that recent surgery had discarded the aspirator and trocar for empyema, except in young children. Free incision was best. He made mention of the operation of exsecting portions of several ribs in old chronic cases.

Dr. Christian, also, thought the danger of air entering was overestimated. He preferred free incision.

Dr. Hadra preferred incision. He mentioned a method of drawing out the fluid under water by means of a syphon arrangement, which is being adopted and is credited to———, although he used it in a case fifteen years ago. Lately hepatic abscesses have been treated in this way with 90% reported cured.

### TYPHLITIS.

Dr. J. H. McCaleb, of Webberville, brought the subject of typhlitis before the Society,* and gave a good discussion of its pathology and treatment. The doctor reported six cases of the milder type occurring in his practice, all successfully treated by opiates, antiseptic enemata, rest, etc. The enemata were given two or three times a day through a tube introduced to the splenic flexure of the colon.

The following case presents unusual interest and an outline of it is given herewith:

### A CAST OF THE VERMIFORM APPENDIX PASSED.

J. J., male, white, age 33, on April 23, 1891, had, as reported, a congestive chill. It proved to be a typical case of typhlitis, and as events proved, caused by foreign bodies in the cæcum, perhaps obstructing the valve, as he passed later undigested food, grape seeds, and a small hard round concretion, the size of a pea; also what resembled musk-melon seed. After this came away the mucous casts.

The symptoms had previously led to diagnosis of and treatment for malarial fever. He was relieved and up to May 2 had no trouble. Then he was seized with a rigor and violent cramping, with temperature of 103½, pulse 110, and respiration 25; tongue coated in center, brownish coat, and raw on the edges. Bowels had not moved since April 27. Intestinal colic violent but paroxysmal. Tenderness in right iliac region, distinct localized tympanitis and gurgling, occasional nausea, and vomited once. Typhlitis was now diagnosed and a hypodermic of sulph. morphia given. In the interval of pain a tube was introduced and colon flushed with a 25% Thierisch's solution as hot as could be borne. Free movements procured and dejections dark grumy mucus and the above "extras." Spirits of turpentine 10 drop doses ordered. Stupes to abdomen. Morphia, as required to relieve pain, one

*In a paper read before the Austin District Medical Society June 25, 1891.

dose at bedtime produced a good night's rest. Bowels moved freely and at 11 a. m., May 4, with the movement came away the mucous membrane of the appendix vermiformis, with considerable tormina. Membrane contained dark mucus—two large beans—several seeds, resembling canteloup seeds, (probably cucumber seeds,) and a small whitish pebble, size of pea. Enemata of hot water continued and more mucous membrane, about an inch of the appendix, passed. Patient recovered.

[This was clearly a case of inflammation from obstruction by foreign bodies. The Doctor says nothing of the circumscribed and movable tumor in right iliac region which characterizes typhlitis.—ED.]

### DISCUSSION ON TYPHLITIS.

Dr. Davis remarked that he was under the impression that foreign bodies would not remain in the system for years, but would ulcerate.

Dr. Wm. Cunningham said that in his experience cases of typhlitis were very rare.

Dr. Fannie Leake reported the case of a young woman, who, when six or seven years old, swallowed a botton, with severe symptoms at the time. For years afterwards she was subject of attacks of severe collicky pains, which attacks could be caused at any time by pressure in the region of the appendix.

Dr. Hadra desired to call attention to an article, by himself, published in the *Annals of Surgery*. (July?) He thought the formation of the tumor was due in some cases to inflammation of the surrounding parts, in others to the accumulation of fœces, and in still others to adhesions of folded up omentum to surrounding parts. There were two clinical types of the disease, he said; the mild, which was usually relieved by the first movement of the bowels, and the severe, which was perhaps always a true disease of the appendix and required prompt surgical interference.

Of the severe form in favorable cases the peritonitis might subside, or the abscess become encapsulated. He mentioned a case in which he operated for the chronic remittent form of the disease. The appendix, which afterwards, on examination, presented evidence of only slight irritation, was in front of the cæcum and turned around. That was the cause of the trouble, he thought, because when the cæcum was full, it lifted the appendix and pulled on the mesentery. He reported three cases, in all of which the appendix was found perforated, and expressed his belief that nearly all the severe cases were due to such a perforation. He advised immediate operation in those cases where the fever is persistent and remittent or has a septic character, and the pulse is getting small and rapid and the patient growing worse. Although the appendix is inside the peritoneum, when the abscess is formed the adhesions shut it off, and by careful operation it can be reached without opening that cavity. He thought the case reported was really an inflammation of the appendix, and that by some favorable chance, instead of leading to perforation, the membrane sloughed away and escaped through the large intestine. Fæcial concretions and foreign bodies located in the appendix were, he thought, rarer than is generally believed.

Dr. Taylor suggested that traumatism might play a part in the etology of typhlitis.

Dr. Cummings made mention of the liability of cases to recur and become worse afterwards.

Adjourned to meet in September, 1891.

T. J. BENNETT, Secretary.

J. W. MCLAUGHLIN, President.

